# Subjective Hunger, Gastric Upset, and Sleepiness in Response to Altered Meal Timing during Simulated Shiftwork

**DOI:** 10.3390/nu11061352

**Published:** 2019-06-15

**Authors:** Charlotte C Gupta, Stephanie Centofanti, Jillian Dorrian, Alison M Coates, Jacqueline M Stepien, David Kennaway, Gary Wittert, Leonie Heilbronn, Peter Catcheside, Manny Noakes, Daniel Coro, Dilushi Chandrakumar, Siobhan Banks

**Affiliations:** 1 Sleep and Chronobiology Laboratory, Behaviour-Brain-Body Research Centre, School of Psychology, Social Work and Social Policy, University of South Australia, 5072 Adelaide, Australia; Stephanie.Centofanti@unisa.edu.au (S.C.); Jill.Dorrian@unisa.edu.au (J.D.); Alison.coates@unisa.edu.au (A.M.C.); Jackie.Stepien-Hulleman@mymail.unisa.edu.au (J.M.S.); Daniel.Coro@mymail.unisa.edu.au (D.C.); siobhan.banks@unisa.edu.au (S.B.); 2 Division of Health Sciences, University of South Australia, 5000 Adelaide, Australia; 3 Robinson Research Institute and Adelaide School of Medicine, University of Adelaide, 5000 Adelaide, Australia; David.kennaway@adelaide.edu.au; 4 Discipline of Medicine, Adelaide Medical School, University of Adelaide, 5000Adelaide, Australia; Gary.wittert@adelaide.edu.au (G.W.); Leonie.heilbronn@adelaide.edu.au (L.H.); 5 South Australia Medical Research Institute (SAHMRI), 5000 Adelaide, Australia; 6 Adelaide Institute for Sleep Health, College of Medicine and Public Health, Flinders University, 5042 Adelaide, Australia; Peter.catcheside@flinders.edu.au; 7 Commonwealth Scientific and Industrial Research Organisation – Food and Nutrition Flagship, 5000 Adelaide, Australia; Mannynoakes@icloud.com; 8 Cognitive Ageing Impairment Neurosciences Laboratory, Behaviour-Brain-Body Research Centre, School of Psychology, Social Work and Social Policy, University of South Australia, 5072 Adelaide, Australia; Dilushi.chandrakumar@mymail.unisa.edu.au

**Keywords:** meal timing, shiftwork, snack, gastric upset, nightshift

## Abstract

Shiftworkers report eating during the night when the body is primed to sleep. This study investigated the impact of altering food timing on subjective responses. Healthy participants (*n* = 44, 26 male, age Mean ± SD = 25.0 ± 2.9 years, BMI = 23.82 ± 2.59kg/m^2^) participated in a 7-day simulated shiftwork protocol. Participants were randomly allocated to one of three eating conditions. At 00:30, participants consumed a meal comprising 30% of 24 h energy intake (Meal condition; *n* = 14, 8 males), a snack comprising 10% of 24 h energy intake (Snack condition; *n* = 14; 8 males) or did not eat during the night (No Eating condition; *n* = 16, 10 males). Total 24 h individual energy intake and macronutrient content was constant across conditions. During the night, participants reported hunger, gut reaction, and sleepiness levels at 21:00, 23:30, 2:30, and 5:00. Mixed model analyses revealed that the snack condition reported significantly more hunger than the meal group (*p* < 0.001) with the no eating at night group reporting the greatest hunger (*p* < 0.001). There was no difference in desire to eat between meal and snack groups. Participants reported less sleepiness after the snack compared to after the meal (*p* < 0.001) or when not eating during the night (*p* **<** 0.001). Gastric upset did not differ between conditions. A snack during the nightshift could alleviate hunger during the nightshift without causing fullness or increased sleepiness.

## 1. Introduction

Shiftwork, characterized by work that occurs outside of regular daytime hours, is increasingly common in today’s society [1]. Many industries, including health care, aviation, transport, and mining, require workers to be available 24/7 [2]. However, shiftwork, particularly nightshift, requires workers to be awake and working at a time that conflicts with their internal circadian clock [3]. Consequently, shiftwork can be associated with a high risk of long-term consequences for the workers, including metabolic disorders, insulin resistance, type 2 diabetes and obesity [4,5], in addition to a higher risk of mood disorders [6,7]. One reason for the health consequences of shiftwork may be that, in a 24 h society, eating becomes a 24 h behaviour [8]. While those on a more traditional day work schedule are most likely to eat three meals per 24 h, with food consumed during the daytime hours [9,10], shiftwork usually leads to altered eating patterns with food consumed across the 24 h period, including at night [8,10,11,12]. Shiftworkers in multiple industries frequently report altered meal timing, with a sample of nurses reporting an increase in altered temporal eating patterns and unbalanced diets compared to day workers [13]. Similarly, miners on rotating shifts report difficulty with following traditional daytime meal patterns [14]. The size of the meal eaten on-shift is also influenced by shiftwork, with an increase in snacking behaviors reported during the nightshift compared to the dayshift in samples of nurses [15], airline crew [16], oil refinery workers [17], and transport workers [18]. 

Eating at night may be of concern given that, during the night, our bodies are primed for sleep [1]. During the night, many digestive processes under complex autonomic, hormonal and circadian control, function differently compared to during the day, including slower rates of gastric emptying [19], gut motility and gastric acid secretion [20], and reduced glucose tolerance [21]. This may play a role in the high rate of long-term gastrointestinal disease that is seen in shiftworker populations, including increased rates of irritable bowel syndrome and peptic ulcers [22], and a high risk of weight-change and obesity [23]. In the short-term, gastric symptoms are commonly reported on-shift by workers [8,24], including disturbed appetite, gastric upset, epigastric pain, gas, and bloating [20]. In numerous studies, these gastric symptoms have been reported during a non-standard shift and linked with eating behaviours. In flight attendants, eating during the nightshift has been associated with increased reporting of stomach aches, bloating, disturbed appetite, diarrhea, and constipation [25,26]. Similarly, in a sample of nurses, certain foods, including core foods, were preferred during the nightshift to reduce digestion issues [27]. This indicates that food choice on-shift may be influenced by the experience of gastric upset at night [11]. 

Reducing the experience of these gastric symptoms on-shift and the long-term burden of metabolic disease on shiftworkers is a priority. A recent laboratory study has shown that not eating during the nightshift and redistributing meals to the daytime hours may limit the metabolic consequences of shiftwork [28]. Further studies have found that consuming a smaller snack compared to a larger snack during the nightshift leads to reduced glucose impairment following breakfast [29], and that eating in the morning to avoid meals during the nightshift reduces insulin levels compared to eating during the night [30]. Similarly, cognitive performance and mood during the nightshift has been shown to be impaired by a large meal compared to not eating during the nightshift and consuming food during the daytime hours [31,32]. Taken together, these findings suggest that altering meal timing leads to improved health and performance outcomes for workers. 

It is also important to consider how endogenous hunger and appetite sensations influence when shiftworkers may eat. A previous laboratory study used 13 days of forced desynchrony consisting of twelve 20 h days with 13 h 20 minutes of wakefulness and 6 h 40 minutes of scheduled sleep, to help separate endogenous and behavioural effects on daily circadian rhythms, with careful control of meals, sleep, activity, posture, temperature, and light [33]. This showed endogenous circadian rhythms in hunger, desire to eat, and appetite, all of which decreased during the night compared to during the day [33]. However, there is very little evidence from controlled studies to help inform whether experiences of hunger, and outcomes of eating, are altered after eating during the nightshift. Only one study has found that participants who did not consume food during the nightshift period reported increased hunger and mild stomach upset compared to those who ate a large meal during the nightshift [32]. 

The lack of specific recommendations about different meal size and meal timing leads to confusion for shiftworkers about which behaviors to adopt. For example, flight attendants have reported choosing to avoid eating while on-nightshift to improve health outcomes [26] whereas paramedics have expressed concern that not eating during the shift could be detrimental to their health [34]. Further, in nurses, reducing meal size during the nightshift has been reported as a strategy for minimizing gastric symptoms during the night [35] and avoiding food altogether has been reported as a strategy to maintain alertness [35]. To extend these results and the recommendations that can be made to workers, alternative meal timing and meal size, including snacking behaviour, should be investigated. The aim of this study was to investigate the impact of eating a large meal, a snack or not eating during simulated nightshifts on hunger, gut reaction, subjective sleepiness, and mood throughout the night. 

## 2. Materials and Methods 

### 2.1. Study Design

The study was an experimental 3-condition between group design and was conducted at the Sleep and Chronobiology Laboratory at the University of South Australia. Twelve study runs were conducted of the 7-day in-laboratory study, that included a 4-day simulated nightshift protocol. All participants gave written informed consent and the study had approval by the University of South Australia Human Research Ethics Committee (#0000033621) and is registered with the Australian New Zealand Clinical Trials Registry (ANZCTR12615001107516). Whilst the primary outcome of the larger study was blood glucose and insulin response to the breakfast meal (to be published elsewhere), this manuscript focusses on the subjective ratings of participants during the night across multiple simulated nightshifts. 

### 2.2. Participants

Healthy, non-shiftworking participants (*n* = 44) were recruited from the general population via flyers and website postings. To determine eligibility, participants were initially telephone screened (age, self-reported height/weight, smoking status, health status, sleep patterns). If eligible, and after informed written study consent, participants then attended two physical screening sessions where they completed questionnaires to determine psychological and physical health, a standard blood test for physical health, wore an activity monitor for 7 days (Phillips Respironics Actiwatch, Murrysville, PA, USA), and completed a 7-day sleep diary to assess habitual sleep patterns and a 4-day food diary to assess habitual eating patterns. Exclusion criteria were the presence of medical, psychiatric disorders, sleep disorders (as confirmed by two commonly used questionnaires assessing the risk for sleep disordered breathing [36,37]), abnormal blood chemistries, habitual sleep duration <7 h or >9 h, BMI outside the normal to overweight range (18.5–27 kg/m^2^), regular medication use (oral contraception was allowed), drug and/or alcohol abuse, methamphetamine abuse, more than 2 h of structured high impact activity/exercise per week, food allergies (or restrained eating), pregnancy, and any trans meridian travel in the 60 days prior to the study, or history of shift-work, to ensure stable sleep/wake patterns and no circadian misalignment. These are standard exclusion criteria for a sleep laboratory study controlling for factors that may influence circadian related behaviours of participants [31,32]. Female participants were only scheduled to participate in the luteal phase of their menstrual cycle, as confirmed by self-report, to control for changes in sleep quality, hormonal factors, and basal metabolism during the menstrual cycle [38,39]. During the 7 days prior to the study, participants refrained from over-the-counter medications, caffeine, alcohol, napping, and were asked to keep a strict habitual sleep schedule (22:00–23:00 to 06:00–07:00), which was verified by actigraphy and a subjective sleep diary. Urine toxicology was performed immediately prior to study commencement to verify the absence of any illicit substances. 

Power analyses were conducted using G*Power [40] based on differences in hunger (ƞ*_p_^2^ =* 0.59), fullness (ƞ*_p_^2^ =* 0.57), thoughts of food (ƞ*_p_^2^ =* 0.54), desire to eat (ƞ*_p_^2^ =* 0.60), bloating (ƞ*_p_^2^ =* 0.30), upset stomach (ƞ*_p_^2^ =* 0.26), dizziness (ƞ*_p_^2^ =* 0.08), and subjective sleepiness (ƞ*_p_^2^ =* 0.23), from our previous studies altering meal timing during simulated shiftwork [31,32]. In order to be sufficiently powered to detect all of these effects in the current three-condition repeated-measures study, we estimated a minimum sample size of 11 participants per group, *n* = 33 participants in total (*a* = 0.05, 1-β = 0.90).

### 2.3. Protocol Design

**Figure 1:**
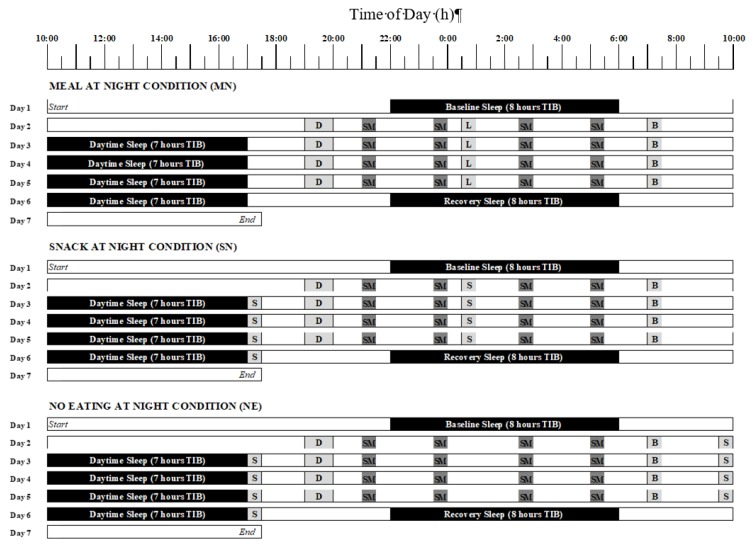
Protocol diagram.

Twelve study runs of the 7-day simulated shiftwork protocol were conducted with 44 participants at the University of South Australia Sleep and Chronobiology Laboratory. This laboratory is windowless, sound attenuated and time-isolated. Ambient temperature was kept at 22 ± 1 °C. For periods of wakefulness, light intensity was <100 lux at eye-level. During periods of sleep, light was fixed at <0.03 lux. Participants had individual bedrooms and bathrooms and spent a total of 7 consecutive days in the laboratory. The protocol of the 7-day study can be seen in Figure 1. Participants entered the laboratory on day 1 and had a baseline sleep on the first night from 22:00 to 06:00. The simulated nightshift protocol began at 06:00 on day 2 and ended at 17:00 on day 6. This nightshift protocol consisted of a simulated nightshift from 20:00 to 0600 (days 2–5; NS1–NS4; Figure 1) and a 7-h daytime sleep opportunity from 10:00 to 17:00 (days 3–6; Figure 1). Prior to the first day shift, participants were kept awake for 28-h. This simulates the period of extended wakefulness commonly reported by shiftworkers prior to the first night in a series of nightshifts [1]. Participants had a recovery sleep opportunity from 22:00 on day 6 to 06:00 on day 7 and then left the laboratory later that day. 

During all wake times, participants were kept awake in the laboratory and continuously monitored by research assistants. Participants completed subjective measurements (see below) at 21:00, 23:30, 2:30 and 5:00 on each day of testing, alone in their individual bedrooms to ensure individual ratings were not influenced by other participants. Previous ratings were not visible to the participants. During other wake times participants had set meals (see Section 2.4), completed cognitive performance tasks (data presented elsewhere), and biological samples including blood, saliva and hair, were collected as part of the aims of the larger study, including metabolic testing. Participants also had free time to read, watch movies, play board games, and interact with other participants and laboratory staff. No vigorous activity was allowed, and there was no access to electronic devices (such as phones or personal computers), live television, or any external time cues. 

Diagram of the 7-day protocol for the in-laboratory simulated shiftwork protocol. The upper panel shows the meal at night condition, the middle panel shows the snack at night condition and the lower panel shows the no eating at night condition. Black bars indicate the sleep periods. Meal times are shown in light grey (D = dinner, B = breakfast, L = lunch [Meal at Night condition], S = snack [Snack at Night condition]). The lunch meal at 00:30 in the Meal at Night condition (upper panel) provided 30% of total 24 h energy intake. The snack at Night condition (middle panel) included a 10% snack at 0030, with the extra 20% of energy redistributed to 17:00. In the No Eating at Night condition (lower panel), no energy was consumed during the nightshift from 20:00 to 6:00 and the 30% meal was redistributed to a 20% snack at 17:00 and a 10% snack at 09:30. Dark shaded boxes indicate when the subjective measures (SM) were recorded (21:00, 23:30, 2:30, 5:00), during which participants performed, in the following order, the Visual Analogue Scales (hunger, fullness, desire to eat, thoughts of food, headache, dizziness, upset stomach and bloating) and the Automated Neurobehavioural Assessment Metric mood scales (subjective sleepiness, fatigue and vigour). 

### 2.4. Eating Conditions

Participants were randomized at the study run level to one of three eating conditions: Meal at Night (MN; 4 runs), Snack at Night (SN; 4 runs), and No Eating at Night (NE; 4 runs). Randomization of run level was done by investigators using an online research randomizer tool (https://www.randomizer.org/). Coding of the conditions was used to blind the investigators during the randomization process and was only broken when data collection occurred. Participants were unaware what time they were eating, did not see meal preparation, and were thus blinded to their eating condition. Further, they ate all meals and snacks in their individual bedrooms and were not influenced by other participants. Participants were also instructed not to talk about food with the other participants during free time. Water was allowed ad libitum during waking periods. The laboratory staff prepared all meals and were not blinded to the eating condition, but avoided all discussions regarding meals with study participants. Twenty-four-hour estimated energy requirement (EER) was calculated for each participant, taking into account the sedentary laboratory environment [41]. EER was rounded to the nearest 500 kJ. The energy requirements were reduced by 15% to allow for the extreme low-level activity laboratory conditions. Energy provided ranged from 8000 to 11,000 kJ/24 h. Regardless of condition, daily 24 h macronutrient content was consistent with the average Australian diet and standardised to approximately 40% carbohydrate, 33% fat, 17% protein and 23 g fibre per 24 h (ABS, 1997). Meal plans were analysed using Foodworks version 8 (Xris Software, Spring Hill, Queensland, Australia) with data sourced from the AusBrands 2017 and AusFoods 2017 databases. On day 1, all participants consumed a lunch meal at 12:00 (30% of 24 h energy intake) and dinner at 19:00 (40% of 24 h energy intake) and all participants consumed a snack on day 7 at 10:00 (10% of 24 h energy intake) and lunch at 12:00 (40% of energy intake). 

**Figure 2:**
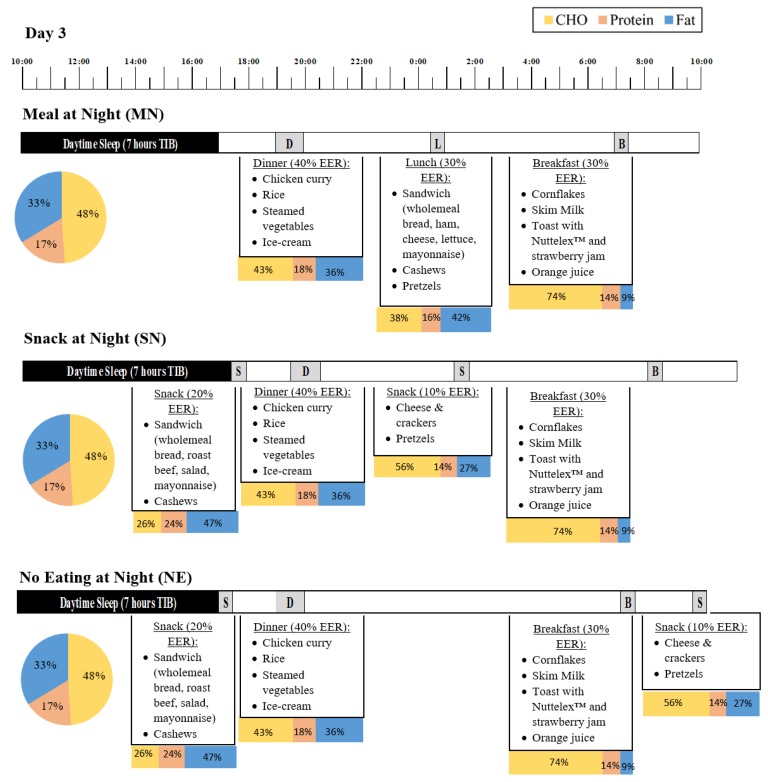
Example meal plan for day 3 of the simulated shiftwork protocol for a participant with an EER of 9500 kJ. The upper panel shows the Meal at Night condition (MN), the middle panel shows the Snack at night condition (SN) and the lower panel shows the No Eating at Night condition (NE). The pie chart represents the proportion of carbohydrates (CHO; yellow), protein (orange), and fat (blue) for the total 24 h period of NS3, and the stacked bars indicate the proportion of carbohydrate, protein, and fat for each meal and snack within the 24 h period of nightshift 3. Values may not total 100% due to small contributions from fibre. There were some variations between conditions in the foods provided, however 24 h macronutrient intake was matched.

During the simulated shiftwork protocol, day 2 to day 6, timing of food intake per 24 h depended on eating condition (Figure 2). Meal timing was altered per 24 h so that the MN condition consumed a meal during the nightshift, the SN condition consumed a snack during the nightshift, and the NE condition did not eat during the nightshift (from 20:00 to 06:00 the NE condition consumed 0% of their 24 h EER). Participants in the MN condition consumed food at 19:00 (dinner, 40% EER), 00:30 (lunch, 30% EER) and 07:00 (breakfast, 30% EER). The SN condition consumed food at 19:00 (dinner, 40% EER), 00:30 (snack, 10% EER), 07:00 (breakfast, 30% EER) and 17:00 (snack, 20% EER). The NE condition consumed food at 19:00 (dinner, 40% EER), 07:00 (breakfast, 30% EER), 09:30 (snack, 10% EER) and 17:00 (snack, 20% EER). Participants had 15 min to complete breakfast, 30 min to complete lunch and snacks, and 45 min to complete dinner meals, and were encouraged by research assistants to consume everything on their plates. On day 2 and day 7, all participants completed Oral Glucose Testing and 10% EER was from a glucose drink. An example menu plan for day 3 of the protocol in each condition, for a participant with an estimated energy requirement of 9500 kJ, can be seen in Figure 2. 

### 2.5. Measures

#### 2.5.1. Hunger and Gut Reaction

Participants completed eight Visual Analogue Scales (VAS) of hunger and gut reaction. The VAS were continuous 10 cm horizontal lines anchored with extremes for each symptom. These were completed by hand by placing a vertical line at the relevant point on the scale. Data were recorded to the nearest millimetre and were double scored by separate scorers to ensure accuracy, with the rescoring of any scales with discrepancies between scorers. The scales completed were hunger (anchored with ‘not at all hungry’ to ‘as hungry as I’ve ever felt’), fullness (‘not at all full’ to ‘as full as I’ve ever felt’), desire to eat (‘very weak desire to eat’ to ‘very strong desire to eat’), thoughts of food (‘no thoughts of food’ to ‘very preoccupied with food’), headache (‘no headache at all’ to ‘extremely bad headache’), dizziness (‘no dizziness’ to ‘a lot of dizziness’), stomach upset (‘no stomach upset’ to ‘extremely upset stomach’), and bloating (‘I don’t feel bloated’ to ‘I feel very bloated’). These scales have been grouped into ‘Food and Hunger’ scales (hunger, fullness, desire to eat, and thoughts of food), and ‘Headache and Gastrointestinal (GI) Symptoms’ scales (headache, dizziness, upset stomach, and bloating). 

#### 2.5.2. Subjective Sleepiness and Fatigue 

Fatigue was assessed using three mood scales from the Automated Neurobehavioural Assessment Metrics test battery (ANAM). Subjective sleepiness was measured using the Stanford Sleepiness Scale-Revised [42], a valid measure of subjective sleepiness [43]. The scale is a 7-point Likert scale of subjective sleepiness (anchored by 1 ‘Feeling very alert, wide awake and energetic’ and 7 ‘very sleepy and cannot stay awake much longer’). Fatigue and vigor were measured from the ANAM mood scale. These two dimensions each consisted of six adjectives rated on a 7-point Likert scale of mood intensity anchored with 0 (not at all) and 6 (very much). The fatigue adjectives were: lazy, inactive, tired, weary, sluggish, and drowsy. The vigour adjectives were: energetic, lively, alert, spirited, active, and vigorous. Each dimension is expressed as the mean score for the 6 adjectives, with higher mean scores for the fatigue dimension indicating more fatigue and higher mean scores for the vigour dimension indicating more vigour. The ANAM mood scales have been validated against the Profile of Mood States and provide a valid assessment of mood [44]. These scales have been grouped into ‘Sleep and Fatigue’ scales (subjective sleepiness, fatigue, and vigour).

#### 2.5.3. Polysomnography and Actigraphy 

Sleep quality and quantity were examined using polysomnography (PSG) during three selected sleep periods: the baseline night sleep opportunity (8 h Time in Bed; TIB), the day sleep on day 5 (7 h TIB) and the recovery night sleep (8 h TIB; Figure 1). Recording was done on a Compumedics Grael EEG amplifier (Compumedics Ltd. Melbourne, Australia). Standard PSG electrode placements were used, and included F3, F4, C3, C4, O1 and O2 sites, with reference to a contralateral mastoid (M1, M2). PSG data were analysed using standard sleep stage scoring criteria [45]. Variables analysed were total sleep time (TST), wake after sleep onset (WASO), sleep efficiency (SE), sleep onset latency (SOL), and the total time in minutes of rapid eye movement (REM), stage 1, stage 2, stage 3, and stage 4 sleep. 

All participants wore two wrist actigraphy monitors (Actiwatch 2, Philips Respironics Inc., Bend, OR, USA) on their non-dominant wrist at all times during the study, except when showering. Two watches were worn to allow for technological failures. The actigraphy monitor is a motion detection sensor that continuously measures physical activity and light exposure. The use of actigraphy as an objective indicator of sleep–wake patterns has been validated with standard measures of sleep and wake states, such as PSG. Variables analysed were TST, SE, and SOL from all sleep periods of the study (baseline, day 3 sleep, day 4 sleep, day 5 sleep, day 6 sleep, and recovery).

#### 2.5.4. Statistical Analysis

Data were screened for outliers before Univariate Analysis of Variance (ANOVA) was used to test for simple differences in age, BMI, average 24 h energy intake and average 24 h macronutrient consumption between groups. Analyses for dependent variables from the VAS (hunger, fullness, desire to eat, thoughts of food, headache, dizziness, upset stomach, and bloating), and the ANAM scales (subjective sleepiness, fatigue, and vigor) were conducted using mixed effects Analysis of Variance (ANOVA) with fixed effects of eating condition (MN, SN and NE), nightshift (NS1-NS4), time of measure (21:00, 23:30, 2:30 and 5:00), and all 2-way and 3-way interaction effects involving condition. Participant ID was included as a random effect to appropriately account for within- and between-subjects variance and variability in individual baseline measurements [46]. Significant interaction effects were further investigated using pre-planned post-hoc comparisons with Bonferroni-adjusted multiple contrasts. Differences in sleep variables (from actigraphy and PSG) were also analysed using mixed-effects ANOVAs, with fixed effects of sleep day (PSG: baseline, day 5 sleep, recovery; Actigraphy (baseline, day 3 sleep, day 4 sleep, day 5 sleep, day 6 sleep, recovery), condition (MN, SN or NE), and an interaction effect of sleep day by condition. There was a random effect of participant ID. Authors conducting the analyses remained blind to eating condition until analyses were completed. Residuals were checked for normality. All analyses were performed using SPSS 22.0 software (IBM Corp, Armonk, NY). Statistical significance was defined as *p* < 0.05. All data are reported as mean ± SD unless otherwise specified. 

## 3. Results

### 3.1. Demographic Information 

**Figure 3:**
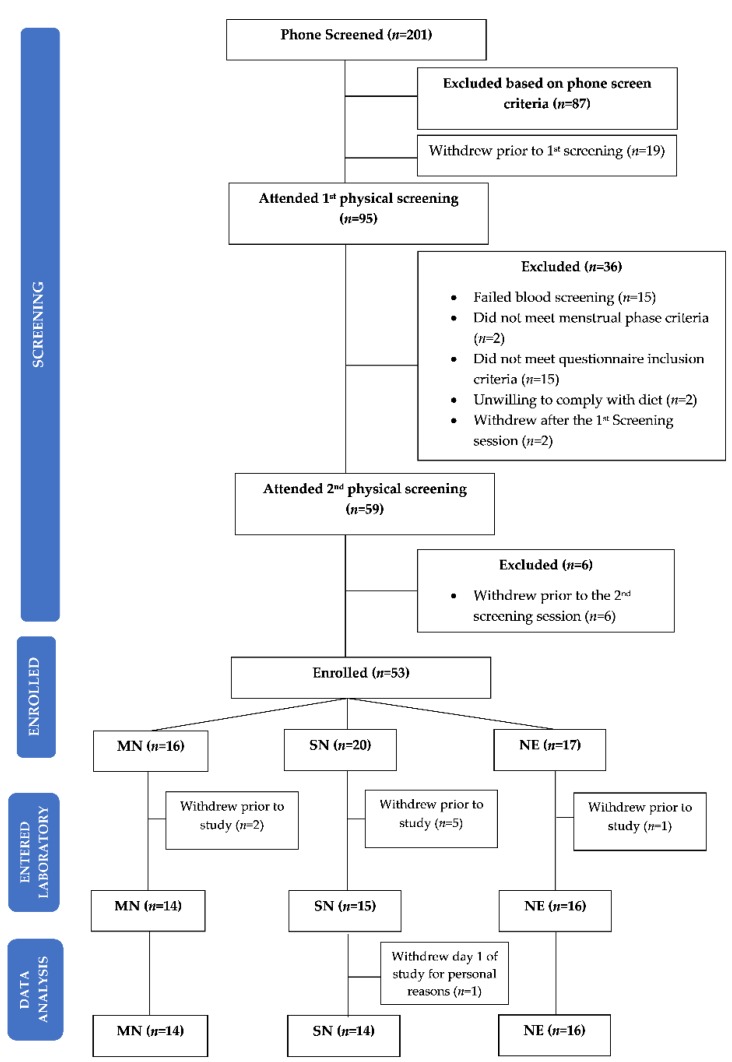
Consort diagram of participants who were screened, enrolled, entered the laboratory, and completed data analysis. MN = Meal at Night condition; SN = Snack at Night condition; NE = No Eating at Night condition.

Fifty-three participants (Figure 3) were randomised by run level to MN (*n* = 16), SN (*n* = 20), and NE (*n* = 17). Between this randomisation and the study beginning, 8 participants (MN *n* = 2; SN *n* = 5; NE *n* = 1) withdrew for personal reasons (*n* = 7) or were withdrawn due to changes in health status that no longer met the inclusion criteria (*n* = 1). Forty-four participants (MN *n* = 14; SN *n* = 14; NE *n* = 16) entered the laboratory, with 1 participant withdrawing from the SN condition on day 1 due to personal reasons. All data from this participant were removed from analysis. All remaining data were reviewed for outliers and no observations were removed from analyses. Within this sample, there was one PSG failure, with PSG data presented for a sample of *n* = 43 (MN *n* = 13; SN *n* = 14; NE *n* = 16), and six participants with two actiwatch failures, with actiwatch data presented for a sample of *n* = 38 (MN *n* = 11; SN *n* = 14; NE *n* = 12). 

Participants in the final sample for analyses (*n* = 44; Table 1) had a mean age of 25.0 ± 4.9 years, mean Body Mass Index (BMI) of 23.8 ± 2.6 kg/m^2^, within the healthy range, and a mean 24 h average energy intake of 9638.2 ± 959.1 kJ. Participant and dietary characteristics for each condition are shown in Table 2. There were no significant differences between conditions in age, BMI, average 24 h energy intake, or macronutrient consumption.

On average, TST for the day sleep was (6.3 ± 0.9 h; Table 1). Participants in all conditions utilized the sleep opportunities equally, with the interaction between condition and sleep period not significant for TST (PSG: F_4, 73.17_ = 0.27, *p* = 0.90; actigraphy F_10, 166.80_ = 0.82, *p* = 0.62), WASO (F_4, 73.11_ = 0.50, *p* = 0.74), SE (PSG: F_4, 73.16_ = 0.28, *p* = 0.89; actigraphy F_4, 164.21_ = 0.62 *p* = 0.80), SOL (PSG F_4, 73.46_ = 0.18, *p* = 0.95; actigraphy F_4, 166.60_ = 0.34 *p* = 0.97), REM (F_4, 72.67_ = 1.02, *p* = 0.40), Stage 1 (F_4, 72.62_ = 0.16, *p* = 0.96), Stage 2, (F_4, 72.28_ = 0.55, *p* = 0.70), Stage 3 (F_4, 72.47_ = 0.65, *p* = 0.63) or Stage 4 (F_4, 71.93_ = 0.42, *p* = 0.79).

### 3.2. Food and Hunger

**Figure 4:**
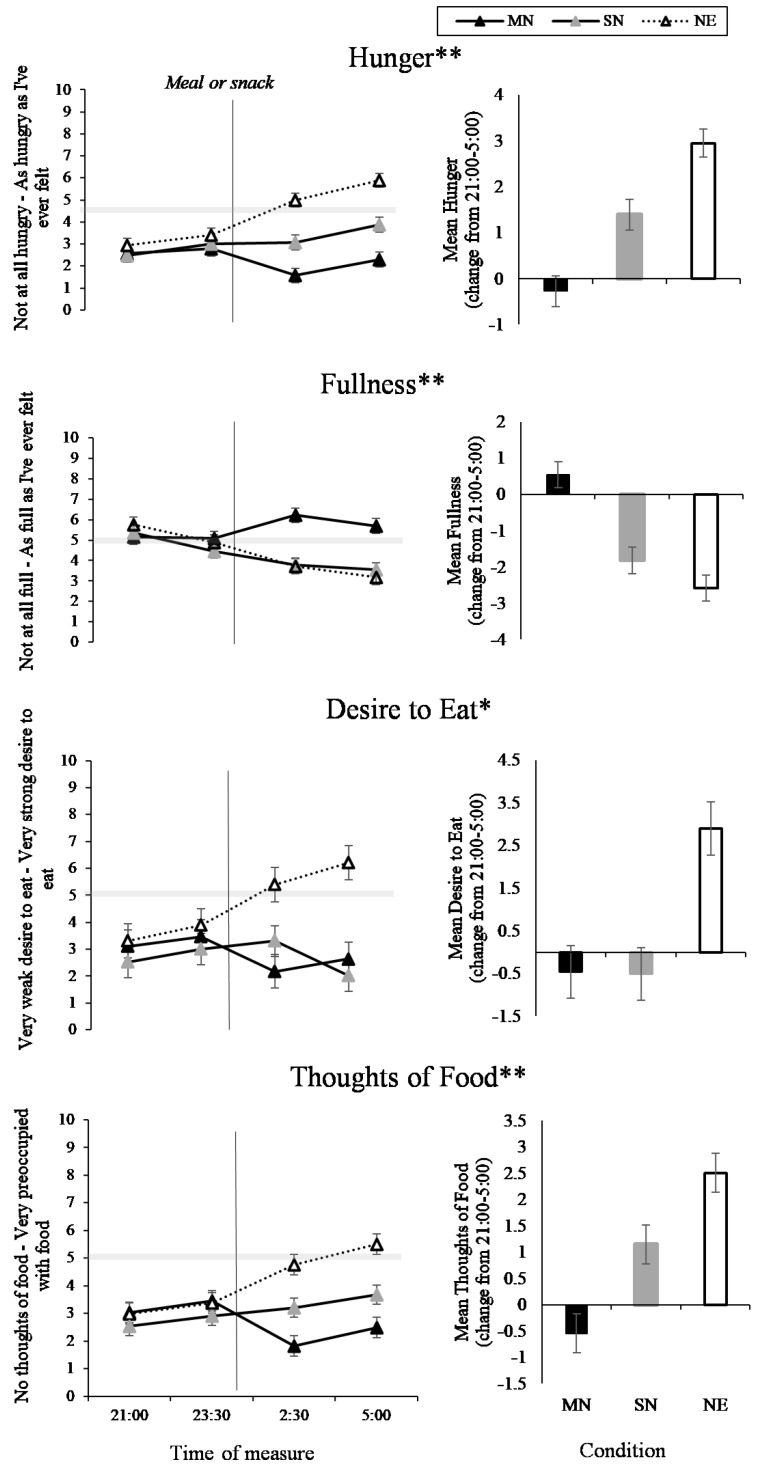
Food and hunger Visual Analogue Scales (VAS) results from the interaction effect between eating condition and time of night (line graphs), and the total mean change from 21:00 to 5:00 (bar graphs). Black lines with black markers and the black columns represent the Meal at Night condition (MN), black lines with grey markers and the grey columns represent the Snack at Night condition (SN), dashed lines with white markers and the white columns indicate the No Eating at Night condition (NE). The vertical black line on each line graph indicates when the meal or snack was consumed. The grey shaded horizontal line on each line graph indicates the midpoint of each VAS. Means presented are estimated marginal means, and error bars indicate standard errors from model estimates. Asterisks indicate a significant interaction effect (*p < 0.05, **p < 0.001).

**Figure 5:**
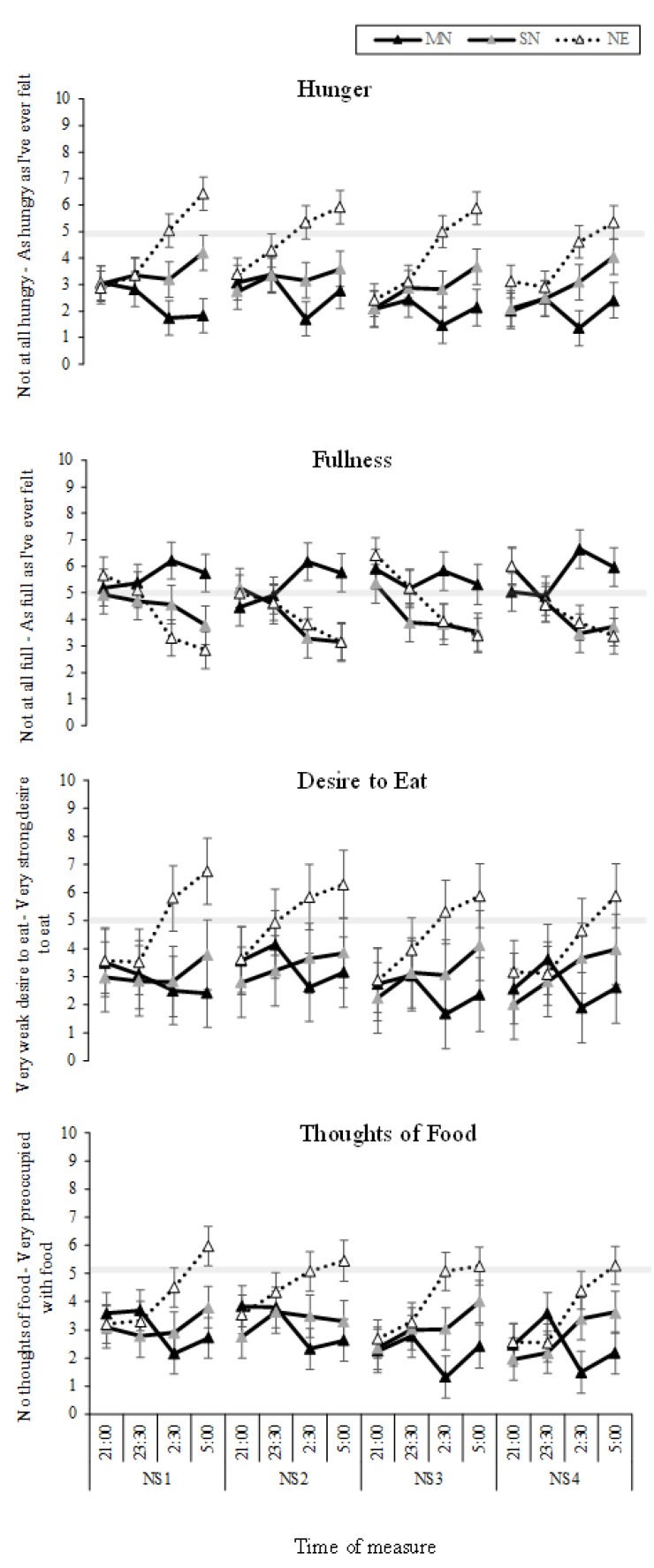
Food and Hunger VAS results from the interaction effect between eating condition by time of night by nightshift (NS1-4). Black lines with black markers represent the Meal at Night condition (MN), black lines with grey markers represent the Snack at Night condition (SN), dashed lines with white markers indicate the No Eating at Night condition (NE). The grey shaded horizontal line on each line graph indicates the midpoint of each VAS. Means presented are estimated marginal means, and error bars indicate standard errors from model estimates.

The interaction between eating condition and time of measure was significant for hunger (*p* < 0.001; Table 1), fullness (*p* < 0.001), desire to eat (*p* = 0.01), and thoughts of food (*p* < 0.001). As can be seen in Figure 4 (left panel), hunger decreased across the nightshift in MN compared to SN (*p* < 0.001) and NE (*p* < 0.001), and hunger increased across the nightshift for SN and NE, with greater hunger in NE (*p* < 0.001). For fullness, the MN condition increased in fullness across the nightshift compared to SN (*p* < 0.001) and NE (*p* < 0.001), and fullness decreased in SN and NE, with no significant difference between SN and NE. Across the night, desire to eat increased in NE, compared to MN (*p* < 0.001) and SN (*p* < 0.001), with no difference between MN and SN (Figure 3). For thoughts of food, Figure 4 shows that thoughts of food increased in NE compared to MN (*p* < 0.001) and SN (*p* < 0.001), with increased thoughts of food in SN compared to MN (*p* = 0.02). The three-way interaction between condition, time of measure, and nightshift was not significant for hunger, fullness, desire to eat or thoughts of food (Figure 5).

### 3.3. Headache and GI Symptoms

**Figure 6:**
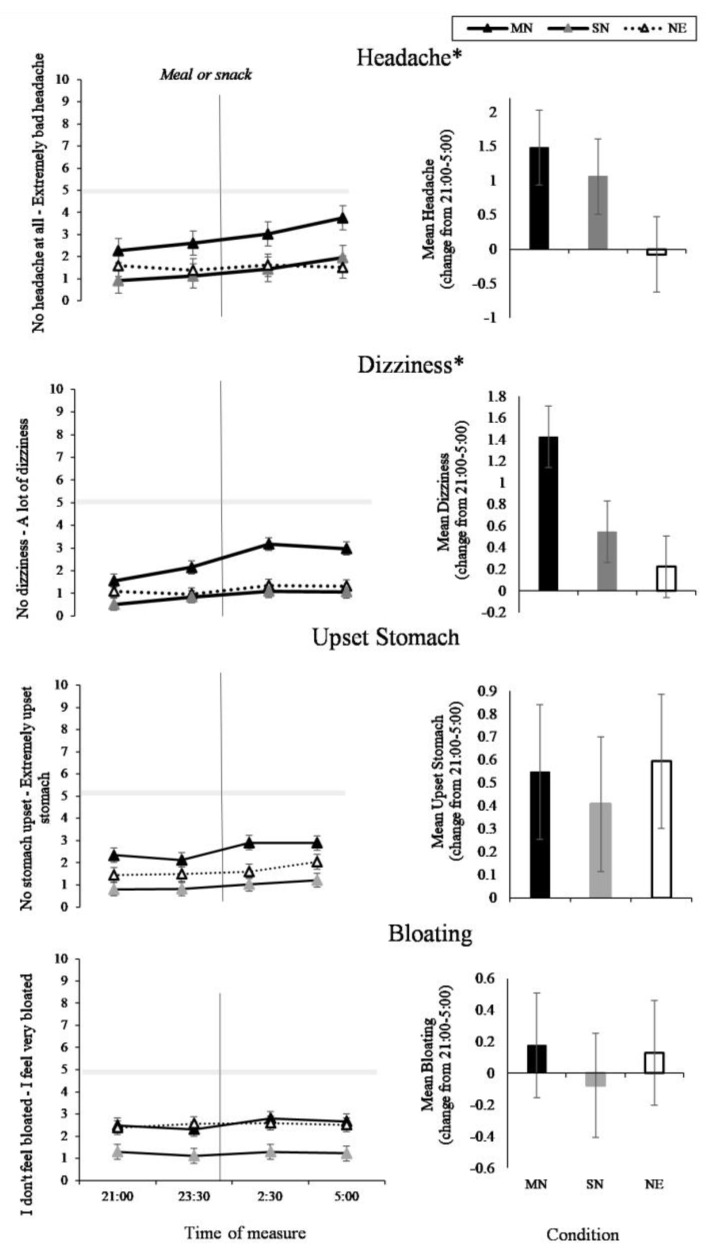
Headache and Gastrointestinal (GI) Symptoms VAS results from the interaction effect between eating condition and time of night (line graphs), and the total mean change from 21:00 to 5:00 (bar graphs). Black lines with black markers and the black columns represent the Meal at Night condition (MN), black lines with grey markers and the grey columns represent the Snack at Night condition (SN), dashed lines with white markers and the white columns indicate the No Eating at Night condition (NE). The vertical black line on each line graph indicates when the meal or snack was consumed. The grey shaded horizontal line on each line graph indicates the midpoint of each VAS. Means presented are estimated marginal means, and error bars indicate standard errors from model estimates. (* *p* < 0.05).

**Figure 7:**
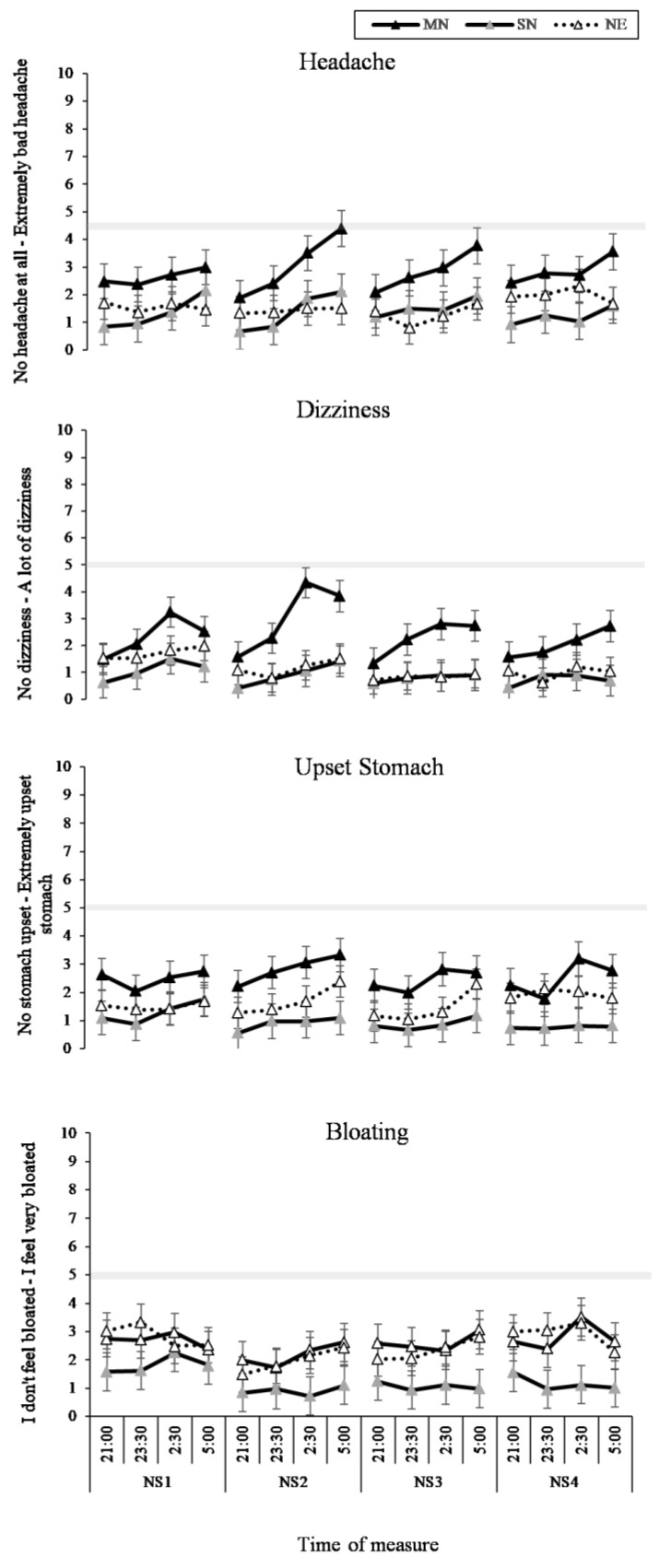
Headache and Gastrointestinal Symptoms (GI) VAS results from the interaction effect between eating condition by time of night by nightshift (NS1-4). Black lines with black markers represent the Meal at Night condition (MN), black lines with grey markers represent the Snack at Night condition (SN), dashed lines with white markers indicate the No Eating at Night condition (NE). The grey shaded horizontal line on each line graph indicates the midpoint of each VAS. Means presented are estimated marginal means, and error bars indicate standard errors from model estimates.

The two-way interaction between time of measure and condition was significant for headache (*p* = 0.01) and dizziness (*p* < 0.01). As can be seen in Figure 6 (left panel), the MN condition had increased reporting of headaches and dizziness compared to the SN condition (*p* = 0.01) and the NE condition (*p* < 0.05), with the greatest change from 21:00 to 5:00 in the MN condition (Figure 6, right panel). The interaction between time of measure and condition was not significant for upset stomach or bloating, and as can be seen in Figure 6, this is likely due to the baseline differences in condition. The three-way interaction between time of measure, condition, and nightshift was not significant for headache, dizziness, upset stomach, or bloating, and, as can be seen in Figure 7, the pattern of difference during the night did not change across the four nightshifts. There was a significant main effect of time of measure on upset stomach (*p* < 0.001).

### 3.4. Sleep and Fatigue

**Figure 8:**
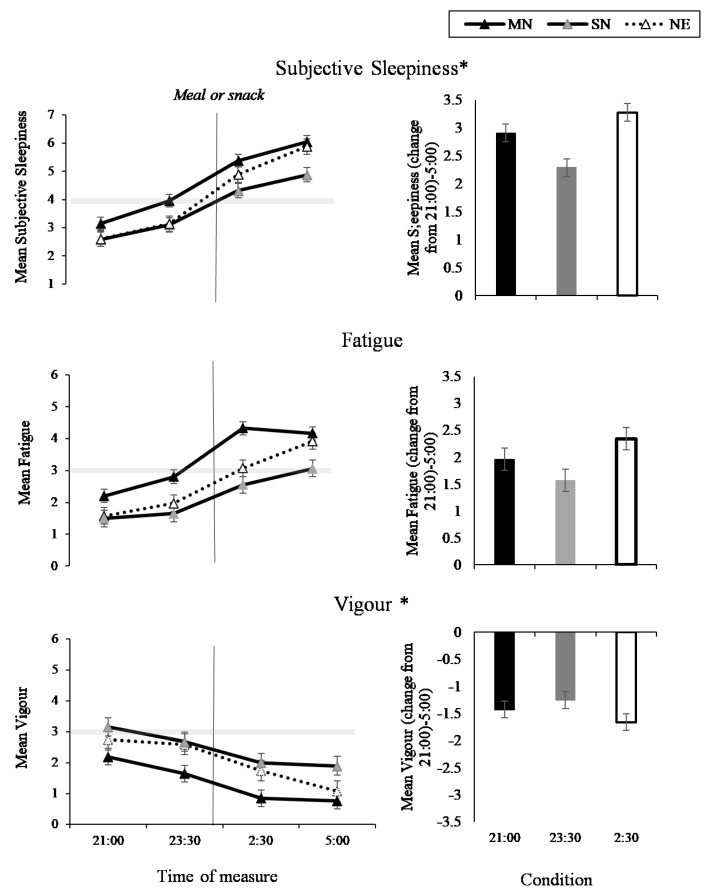
Sleep and fatigue results from the interaction effect between eating condition and time of night (line graphs), and the total mean change from 21:00 to 5:00 (bar graphs) for sleepiness, fatigue, and vigour. Black lines with black markers and the black columns represent the Meal at Night condition (MN), black lines with grey markers and the grey columns represent the Snack at Night condition (SN), dashed lines with white markers and the white columns indicate the No Eating at Night condition NE). The vertical black line on each line graph indicates when the meal or snack was consumed. The grey shaded horizontal line on each line graph indicates the midpoint of each VAS. Means presented are estimated marginal means, and error bars indicate standard errors from model estimates. Asterisks indicate a significant interaction effect (* *p* < 0.05).

**Figure 9:**
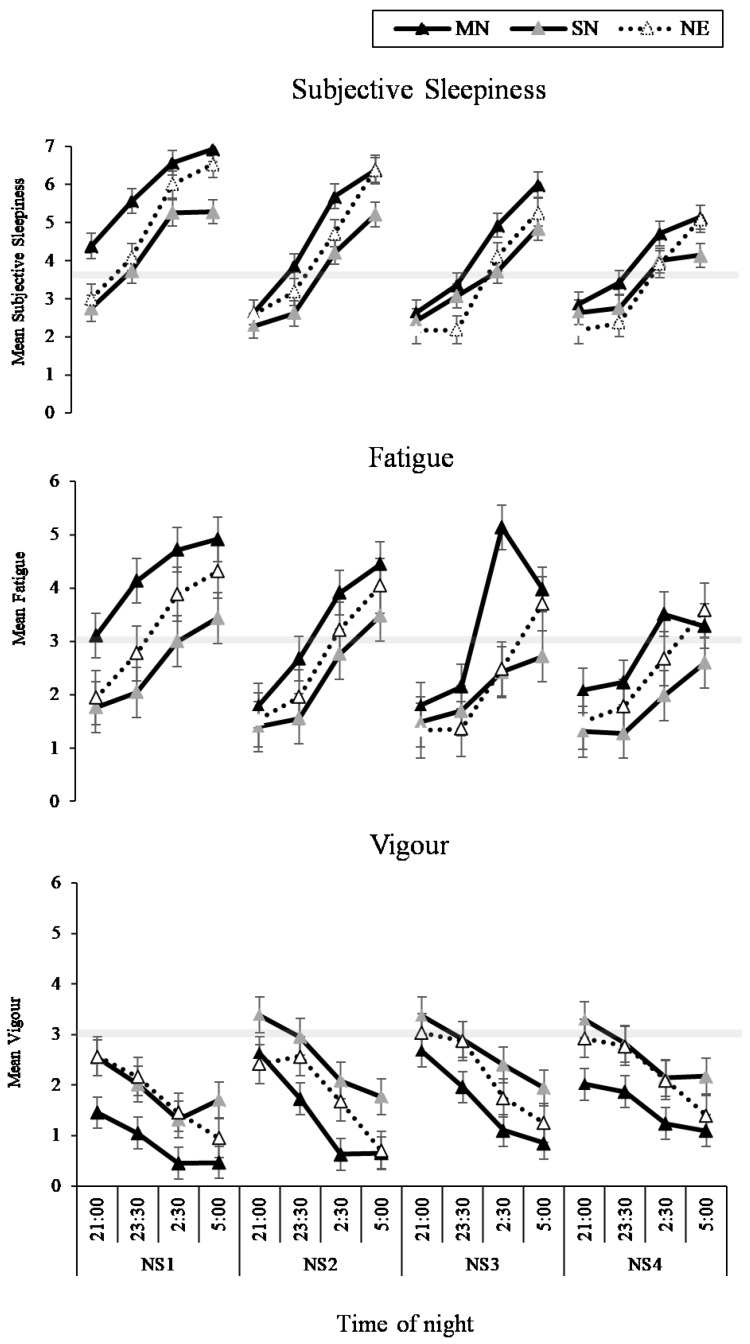
Sleep and fatigue results from the interaction effect between eating condition by time of night by nightshift (NS1-4) for sleepiness, fatigue and vigour. Black lines with black markers represent the Meal at Night condition (MN), black lines with grey markers represent the Snack at Night condition (SN), dashed lines with white markers indicate the No Eating at Night condition (NE). The grey shaded horizontal line on each line graph indicates the midpoint of each VAS. Means presented are estimated marginal means, and error bars indicate standard errors from model estimates.

The interaction between condition and time of measure was significant for sleepiness (*p* < 0.001) and vigour *(p =* 0.01), such that sleepiness increased and vigour decreased in all conditions across the night (Figure 8, left panel), with the least amount of change in the SN condition (Figure 8, right panel). While the pattern of change was similar, the two-way interaction between condition and time of measure was not significant for fatigue, (*p* = 0.06, Figure 8). The three-way interaction between condition, time of measure, and nightshift was not significant for sleepiness, fatigue or vigour (Figure 9). The main effects of condition, time of measure, and nightshift were significant for fatigue (*p* < 0.05).

## 4. Discussion

This is the first study to investigate the impact of consuming either a meal, a snack, or not eating, during four simulated nightshifts, on hunger, gut reaction, and sleepiness. Ratings of hunger, fullness and thoughts of food followed a dose-dependent response to the eating conditions, with the greatest hunger and least amount of fullness and thoughts of food in the meal at night condition. When considering the increases in sleepiness, headaches and dizziness across the night, those who ate the snack reported the smallest increases in all of these measures.

Participants who consumed a meal during the nightshift reported feeling less hungry, less desire to eat, less thoughts of food, and increased fullness compared to those who consumed the snack or did not eat during the nightshift. This is as expected, given that the food intake pattern of the meal condition provided 20–30% more of their daily EER to be distributed during the night compared to the other conditions. Those who did not eat during the nightshift were significantly hungrier and had greater desire to eat and thoughts of food compared to those who ate the large meal, however they did not report extreme levels of hunger. Whilst this may predominantly reflect a longer inter-meal interval, hunger displays a circadian rhythm and is decreased during the night [33] and could also play a role. One potential mechanism for this effect could be levels of leptin and ghrelin during the night. Leptin has been shown to exhibit a nocturnal rise and ghrelin a nocturnal decrease [47,48]; this corresponds with our results showing no extreme levels of hunger during the nightshift. However, shiftworkers have reported increasing hunger across nightshifts and describe hunger as a factor influencing their decision to eat during the nightshift [8,35]. Leptin and ghrelin may potentially be influenced by sleep restriction [49,50] and sleep deprivation [47]. This has also been shown to influence meal choice. In a laboratory study in which sleep was restricted to a 4 h sleep opportunity per 24 h, the odds of choosing to eat a snack were greater than in the moderate or severe sleep restriction conditions [51]. While leptin and ghrelin levels were not measured in the current study, participants had a 7 h sleep opportunity after each nightshift and were therefore not sleep restricted across the nightshift protocol. Many shiftworkers are chronically sleep restricted and have sleep that is shortened by 2–4 h compared to traditional day workers [52]. Further, this sleep is often disrupted by external factors such as light and environmental noise [1]. This may result in increased hunger during the nightshift for shiftworkers in the real world who are working multiple shifts and not receiving adequate sleep, particularly across consecutive shifts when cumulative sleep loss may occur [1].

Participants who consumed a snack during the nightshift were less hungry and had less thoughts of food than those who did not eat during the night, while also reporting less fullness than the meal at night group. This was expected given that they consumed less food during the time the ratings were captured. Interestingly, there was no difference in desire to eat between those who had a snack and those who had a meal. This suggests that although the snack did not lead to feelings of fullness, participants did not want to eat more after eating the snack. Given the recent research highlighting the potential metabolic consequences of eating a large meal during the night [28,30], a small snack, representing only 10% of daily EER, may be a more appropriate and realistic option for shiftworkers. Indeed, snacking is frequently reported by shiftworkers, with many shiftworkers reporting consuming small portions of food during the nightshift, such as muesli bars, nuts, chips, and cookies [15,35,53].

Studies in shiftworkers, such as paramedics, suggest a level of concern that not eating during the nightshift may lead to poor health [34]. However, that notion is challenged by the current findings suggesting reductions in physical symptom reporting with reduced food consumption during the night. There was an impact of meal timing on ratings of headaches and dizziness, with greater headaches and dizziness reported after eating the meal compared to after the snack or not eating. Studies suggest that shiftworkers report headaches and dizziness on shift when they work overnight [22]. The current findings suggest that altering meal timing to avoid large meals during the nightshift and consuming a snack or not eating could potentially reduce these symptoms overnight. However, it is important to note that ratings of headaches and dizziness symptoms remained quite low. Future research should further investigate the relationship between meal timing during the nightshift and physical symptoms, such as headaches and dizziness. Interestingly, we found no difference in stomach upset or bloating based on meal timing. Participants who consumed a snack or a meal during the night consumed food when gut function is typically at its lowest [19,54]. Given that shiftworkers report an increase in gut related symptoms in response to eating during the nightshift [26,27], we expected to find an increase in reports of gut-related symptoms in the group who ate a large meal during the night. In the current study, food was only consumed on one occasion, at 00:30, and perhaps in the real world shiftworkers who report gastric upset are consuming greater amounts of food and eat later in the shift, nearer to the trough of gut function [19]. Further, one study found that nurses split meals into several snacks consumed across the shift [35]. This may influence gut reaction differently to one snack or meal consumed at the beginning of the shift. Shiftworkers also often report consuming foods at night that are high in fat and sugar, including cookies, chocolate, cake, and biscuits [10,17,54], whereas in the current study foods were lower in fat and higher in carbohydrate content. Perhaps the foods that are higher in fat have a greater impact on gastric upset during the nightshift. Additionally, while pre-study food diaries were used to ensure that the study diet was not substantially different from the participants’ typical food intake, there may have been some changes for all participants. This could have masked some differences between conditions for gastric upset. Future research should be mindful of using pre-study diets.

Regardless of eating condition, all participants reported increased sleepiness and fatigue, and decreased vigour, across the nightshift. This is expected due to increasing circadian sleep pressure and decreasing alertness across the night [1]. We found that consuming a snack during the nightshift attenuates the increase in sleepiness and decreases in vigour across the shift relative to consuming a meal or not eating during the night. Consistent with our previous research, we found that sleepiness increased across the nightshift but did not differ between those that had a large meal and those that did not eat during the night [31]. Given that many shiftworkers eat during the nightshift [15,35] the current research has furthered this previous result and showed that shiftworkers may be able to eat during the night without feeling increased sleepiness, however this should be a small snack and not a large meal. During the day, post-prandial increases in sleepiness are commonly reported, with greater sleepiness after a lunch meal [55,56,57]. It is important to note that post-lunch, individuals typically experience an increase in sleep pressure [58], and therefore a lunch meal potentially exacerbates sleepiness during the day. Consuming the large meal may similarly be exacerbating subjective sleepiness during the night. Of note, in the daytime studies showing post-prandial sleepiness after lunch, the meals were high in carbohydrate [55,56]. However, in the current study the snack consumed during the night was higher in carbohydrate than the meal consumed at night (for example, on day 3, the meal consumed during the nightshift was 38% carbohydrate and the snack at night was 56%; Figure 2). This suggests that the post-prandial effects of different macronutrients can differ based on time of day.

Shiftworkers often report that one of the main factors influencing why they eat on shift is to increase alertness [11]. In a sample of nurses, dietary strategies and altering eating behaviours were reported as a strategy to maintain alertness during the nightshift [59]. Similarly, a sample of oil refinery workers reported snacking on savory and sweet snacks while on-shift to improve alertness [17] and, in the aviation industry, pilots and air stewards report snacking for the same reasons [60]. Given that participants in the current study received a 7 h day sleep opportunity after each nightshift, sleep restriction was likely minimal. However, in the real world, shiftworkers must cope with factors including environmental disruptions and family commitments, all of which may impact their sleep quantity and quantity [1], and consequently may lead to increased sleepiness in response to food consumed at night. Given that sleepiness and fatigue are frequently reported by shiftworkers as influencing their eating behaviours, more research is needed to understand the impact of altered meal timing on sleepiness.

This study represents an important first step to investigate the specific impact of altered meal timing during the nightshift. There are two deliberate design elements of this study that preserve experimental control, but limit real-world generalizability. First, this study was conducted in a controlled laboratory environment, with controlled sound, lighting, temperature, and no environmental timing cues, and was conducted with a sample of young and healthy individuals. The sleep laboratory was an ideal sleep environment and participants (screened to ensure that they were ‘good’ sleepers) were allowed a 7 h sleep opportunity after each nightshift. All participants utilized this sleep opportunity, with no differences between eating condition in sleep quality or quantity. This limits direct generalizability to shiftworkers, who may be experiencing chronic sleep restriction [1], which could lead to altered responses to food intake during the night [61]. Further studies where daytime sleep is restricted would be of benefit. Second, shiftworkers were not recruited in this study, as this is a group that will commonly experience circadian misalignment and disrupted sleep/wake patters [1], which may further exacerbate subjective symptoms explored in this study. Further, the generalizability of the results will be strengthened when samples reflect shiftworker populations more closely. This includes older participants and those with habits such as smoking [62], in addition to the psychological and physical health issues that are especially common among shiftworkers, including obesity [63], metabolic disorders [4,64], sleep disorders [65], and mood disorders [66,67]. Further, shiftworkers report frequent caffeine use whilst on shift [68,69] and this is known to influence gastric upset [70]. These factors were controlled in the current study, however these health factors may be influencing both eating habits and the biological response to food that shiftworkers experience [71], potentially worsening the impact of food on subjective reactions during the night.

To overcome these limitations and build on these findings, there are several recommendations for future research. While altering meal timing to avoid consuming a meal during the nightshift should be investigated in real shiftwork environments with shiftworkers, there are also a number of laboratory studies that will allow for greater investigation of these variables. In the current study, participants consumed one meal or snack during the nightshift, however we know that shiftworkers often snack multiple times throughout the shift [9,53]. Investigating the impact of multiple snacks throughout the nightshift, especially later in the night is important, particularly given hunger reported by those who did not eat during the nightshift peaked at 5:00. Further, investigating different macronutrient balances is important for understanding the impact of eating at night on gut reaction, as shiftworkers typically report consuming foods high in sugar and fat, including chips, chocolate, and fast food [11]. To recommend eating patterns most applicable to shiftworkers, it is important to understand the factors that influence eating behaviour [11]. To understand whether altering food timing to consume no food or a small snack during the nightshift is viable in various shiftworker contexts, qualitative methods, such as interviews, will be necessary.

## 5. Conclusion 

Altered meal timing is important to consider in the goal of improving health and wellbeing during the night, in addition to reducing long-term gastrointestinal upset. This study is the first to suggest a differential impact of eating a meal, snack or not eating during the nightshift on hunger, headache and GI symptoms, sleepiness, and fatigue during the nightshift. While future research is needed to build on these results, our findings suggest that a small snack during the night may protect shiftworkers from the increased sleepiness experienced after a large meal and the hunger experienced when not eating during the night.

## Figures and Tables

**Table 1:** Results from the mixed effects Analysis of Variance (ANOVA) showing the main effects of eating condition (MN, SN, NE), Time of measure (21:00, 23:30, 2:30, 5:00) and Nightshift (NS1-NS4), and the interaction effects of eating condition by time of measure, eating condition by nightshift, and eating condition by time of measure by nightshift.

	Eating Condition		Time of Measure		Nightshift		Eating Condition* Time of Measure		Eating Condition* Nightshift		Eating Condition* Time of Measure* Nightshift	
	F_(df)_	*p*	F_(df)_	*p*	F_(df)_	*p*	F_(df)_	*p*	F_(df)_	*p*	F_(df)_	*p*
Food and Hunger												
Hunger	4.95_(2, 41.07)_	0.12	19.58_(3, 626.15)_	**<0.001**	3.99_(3, 626.27)_	**0.01**	15.85_(6, 626.15)_	**<0.001**	0.33_(6, 626.27)_	0.92	0.41_(18, 626.15)_	0.99
Fullness	1.80_(2, 41.09)_	0.18	15.78_(3, 627.16)_	**<0.001**	0.86_(3, 627.26)_	0.46	12.16_(6, 627.16)_	**<0.001**	0.41_(9, 627.26)_	0.87	0.58_(18, 627.16)_	0.92
Desire to Eat	4.08_(2, 41.16)_	**0.02**	1.06_(3, 627.46)_	0.37	0.22_(3, 627.90)_	0.88	3.80_(6, 627.46)_	**0.01**	1.46_(6, 627.91)_	0.19	1.08_(18, 627.47)_	0.37
Thoughts of Food	2.15_(2, 41.03)_	0.13	9.72_(3, 627.09)_	**<0.001**	4.34_(3, 627.19)_	**0.01**	13.71_(6, 627.09)_	**<0.001**	0.67_(6, 627.19)_	0.67	0.42_(18, 627.10)_	0.98
Headache and Gastrointestinal Symptoms												
Headache	2.73_(2, 41.01)_	0.08	10.59_(3, 630.05)_	**<0.001**	0.20_(3, 630.11)_	0.90	3.06_(6, 630.05)_	**0.01**	1.24_(6, 630.11)_	**0.01**	0.45_(18, 630.05)_	0.98
Dizziness	3.81_(2, 41.07)_	**0.03**	11.87_(3, 630.11)_	**<0.001**	6.41_(3, 630.19)_	**<0.001**	3.39_(6, 630.12)_	**0.003**	2.33_(6, 630.19)_	**0.03**	0.39_(18, 630.12)_	0.99
Upset Stomach	3.21_(2, 41.02)_	0.05	5.87_(3, 627.07)_	**<0.001**	0.98_(3, 627.14)_	0.40	0.55_(6, 627.07)_	0.77	1.37_(6, 627.14)_	0.23	0.41_(18, 627.07)_	0.99
Bloating	1.98_(2, 41.02)_	0.15	0.62_(3, 629.06)_	0.60	8.90_(3, 629.11)_	**<0.001**	0.50_(6, 629.06)_	0.81	0.85_(6, 629.11)_	0.53	0.65_(18, 629.06)_	0.86
Sleep and Fatigue												
Subjective Sleepiness	4.50_(2, 40.90)_	**0.02**	359.94_(3, 596.06)_	**<0.001**	80.35_(3, 596.10)_	**<0.001**	4.12_(6, 596.05)_	**<0.001**	3.37_(6, 596.09)_	**<0.01**	0.83_(18, 596.06)_	0.66
Fatigue	6.01_(2, 41.00)_	**0.01**	63.89_(3, 615.00)_	**<0.001**	14.27_(3, 615.00)_	**<0.001**	2.05 _(6, 615.00)_	0.06	1.19_(6, 615.00)_	0.31	0.69_(18, 615.00)_	0.82
Vigour	3.95_(2, 41.00)_	**0.03**	119.19_(3, 615.00)_	**<0.001**	25.03_(3, 615.00)_	**<0.001**	2.72_(6, 615.00)_	**0.01**	1.52_(6, 615.00)_	0.17	0.70_(18, 615.00)_	0.81

Note. MN: Meal at Night; SN: Snack at Night; NE: No Eating at Night; NS: Nightshift, bold indicates a significant value (*p* < 0.05), F-statistic: variation between sample means, df: degrees of freedom.

**Table 2:** Participant sex, age, BMI, average 24 h energy intake, and average 24 h macronutrient consumption.

Variable	MN	SN	NE	All
*n* (%male)	14 (57%)	14 (57%)	16 (63%)	44 (59%)
Age (years)	23.3 ± 3.7	25.4 ± 5.7	24.9 ± 2.6	25.0 ± 4.9
BMI (kg/m^2^)	23.9 ± 2.8	23.9 ± 2.8	23.7 ± 2.4	23.8 ± 2.6
Average 24 h energy intake (kJ)	9416.9 ± 961.0	9300.0 ± 942.3	9392.3 ± 1028.8	9638.2 ± 959.1
*Average 24 h macronutrient consumption*				
Carbohydrate (g)	267.9 ± 24.9	267.3 ± 25.2	269.7 ± 25.6	268.4 ± 25.1
Protein (g)	93.2 ± 8.7	91.3 ± 9.9	92.0 ± 10.4	92.1 ± 9.7
Fat (g)	86.9 ± 11.6	84.6 ± 12.7	85.2 ± 12.8	85.6 ± 12.4
Fibre (g)	27.4 ± 3.0	26.0 ± 2.5	26.8 ± 2.5	26.5 ± 2.7
*Polysomnography*				
*TST (h)*				
Baseline (8 h)	7.2 ± 0.7	7.0 ± 0.5	7.19 ± 0.4	7.1 ± 0.5
Day 5 recovery sleep (7 h)	6.4 ± 0.9	6.3 ± 0.6	6.3 ± 0.6	6.2 ± 0.9
Recovery (8 h)	6.1 ± 0.9	5.8 ± 1.3	6.2 ± 1.3	5.8 ± 1.5
*WASO (min)*				
Baseline (8 h)	25.71 ± 26.51	27.68 ± 16.89	29.00 ± 13.91	27.39 ± 19.64
Day 5 recovery sleep (7 h)	32.31 ± 53.80	34.18 ± 35.33	37.54 ± 34.74	33.81 ± 40.94
Recovery (8 h)	57.85 ± 65.72	66.50 ± 56.61	50.75 ± 45.34	72.66 ± 82.80
*SE (%)*				
Baseline (8 h)	90.09 ± 8.31	87.61 ± 6.46	90.25 ± 4.51	89.27 ± 6.65
Day 5 recovery sleep (7 h)	91.07 ± 13.10	90.31 ± 9.01	89.81 ± 8.27	90.56 ± 10.01
Recovery (8 h)	76.13 ± 12.11	73.04 ± 15.90	76.92 ± 15.90	72.78 ± 18.11
*SOL (min)*				
Baseline (8 h)	21.68 ± 15.49	31.68 ± 24.06	17.67 ± 11.69	23.98 ± 18.63
Day 5 recovery sleep (7 h)	5.31 ± 3.33	6.57 ± 3.82	5.29 ± 2.51	5.71 ± 3.24
Recovery (8 h)	56.77 ± 35.52	62.69 ± 59.72	60.17 ± 85.24	57.95 ± 60.20
*REM (min)*				
Baseline (8 h)	103.43 ± 29.54	87.86 ± 15.94	94.5 ± 22.07	95.30 ± 23.60
Day 5 recovery sleep (7 h)	107.81 ± 29.55	86.54 ± 18.75	104.71 ± 19.91	98.04 ± 25.40
Recovery (8 h)	88.46 ± 23.93	84.92 ± 25.63	88.08 ± 30.34	83.91 ± 29.15
*Stage 1 (min)*				
Baseline (8 h)	5.64 ± 2.78	6.93 ± 5.48	4.96 ± 3.67	5.89 ± 4.13
Day 5 recovery sleep (7 h)	5.42 ± 2.96	6.25 ± 3.53	4.83 ± 3.54	5.41 ± 3.37
Recovery (8 h)	6.58 ± 3.94	8.92 ± 5.74	6.83 ± 5.69	7.46 ± 5.17
*Stage 2 (min)*				
Baseline (8 h)	157.25 ± 52.22	158.54 ± 60.93	151.92 ± 39.35	156.10 ± 50.90
Day 5 recovery sleep (7 h)	111.23 ± 30.86	121.79 ± 46.76	109.88 ± 38.27	112.38 ± 40.77
Recovery (8 h)	159.50 ± 46.17	140.77 ± 56.39	153.79 ± 49.17	146.69 ± 53.23
*Stage 3 (min)*				
Baseline (8 h)	79.68 ± 25.25	80.75 ± 33.48	89.08 ± 38.64	82.88 ± 32.01
Day 5 recovery sleep (7 h)	65.92 ± 20.97	63.96 ± 22.21	61.13 ± 22.66	62.90 ± 21.84
Recovery (8 h)	63.54 ± 21.93	66.54 ± 48.97	68.08 ± 38.02	63.98 ± 37.11
*Stage 4 (min)*				
Baseline (8 h)	86.36 ± 32.80	86.18 ± 49.50	91.00 ± 22.24	87.69 ± 36.33
Day 5 recovery sleep (7 h)	91.62 ± 40.20	100.11 ± 44.91	97.21 ± 29.37	96.05 ± 37.82
Recovery (8 h)	47.42 ± 23.08	48.88 ± 24.51	52.67 ± 25.03	47.21 ± 25.28
*Actigraphy*				
*TST (h)*				
Baseline (8 h)	7.8 ± 0.5	7.2 ± 1.3	7.8 ± 0.1	7.9 ± 0.5
Day 3 sleep (7 h)	6.9 ± 0.1	6.7 ± 0.7	6.9 ± 0.1	6.9 ± 0.1
Day 4 sleep (7 h)	6.7 ± 0.6	6.7 ± 0.4	6.9 ± 0.1	6.8 ± 0.2
Day 5 sleep (7 h)	6.9 ± 0.1	6.7 ± 0.4	6.9 ± 0.1	6.8 ± 0.3
Day 6 sleep (7 h)	6.7 ± 0.5	6.8 ± 0.1	6.9 ± 0.1	6.8 ± 0.3
Recovery (8 h)	7.6 ± 0.8	7.9 ± 0.1	7.7 ± 0.5	7.7 ± 0.4
*SE (%)*				
Baseline (8 h)	83.00 ± 14.52	85.73 ± 11.30	90.67 ± 5.42	87.80 ± 9.99
Day 3 sleep (7 h)	86.98 ± 9.70	88.87 ± 10.00	90.88 ± 4.18	89.65 ± 7.10
Day 4 sleep (7 h)	83.90 ± 14.18	85.76 ± 10.42	90.74 ± 4.68	87.28 ± 10.63
Day 5 sleep (7 h)	84.91 ± 13.80	87.54 ± 10.88	91.30 ± 3.40	87.88 ± 10.66
Day 6 sleep (7 h)	84.28 ± 13.36	88.30 ± 7.63	89.48 ± 4.90	87.56 ± 9.59
Recovery (8 h)	77.25 ± 15.62	83.81 ± 10.84	85.57 ± 9.69	83.30 ± 11.48
*SOL (min)*				
Baseline (8 h)	5.85 ± 10.37	8.00 ± 12.20	5.17 ± 4.61	5.65 ± 9.08
Day 3 sleep (7 h)	2.54 ± 5.03	2.5 ± 4.91	1.25 ± 1.322	1.76 ± 3.36
Day 4 sleep (7 h)	1.31 ± 2.45	4.18 ± 5.52	2.75 ± 3.14	2.65 ± 4.10
Day 5 sleep (7 h)	1.38 ± 2.26	2.12 ± 3.17	2.58 ± 2.92	2.04 ± 2.83
Day 6 sleep (7 h)	3.23 ± 7.65	3.86 ± 5.16	2.54 ± 1.90	2.66 ± 3.59
Recovery (8 h)	13.27 ± 24.64	7.21 ± 21.16	6.23 ± 5.65	7.23 ± 12.18

Note: Data presented as M ± SD. MN: Meal at Night; SN: Snack at Night; NE: No Eating at Night. PSG *n* = 43 (MN *n* = 13; SN *n* = 14; NE *n* = 16), actigraphy *n* = 38 (MN *n* = 11; SN *n* = 14; NE *n* = 12). TST: total sleep time, WASO: wake after sleep onset, SE: sleep efficiency, SOL: sleep onset latency, REM: rapid eye movement sleep, min: minute, %: percentage.
